# An exploratory study into the effect of time-restricted internet access on face-validity, construct validity and reliability of postgraduate knowledge progress testing

**DOI:** 10.1186/1472-6920-13-147

**Published:** 2013-11-06

**Authors:** Marja GK Dijksterhuis, Izabela Jozwiak, Didi DM Braat, Fedde Scheele

**Affiliations:** 1 Department of Obstetrics and Gynecology, Amphia Ziekenhuis, Langendijk 75, 4819 EV, Breda, The Netherlands; 2 Management of Learning, Maastricht University School of Business and Economics, Tongersestraat 53, 6211 LM, Maastricht, The Netherlands; 3 Department of Obstetrics and Gynecology, University Medical Center Nijmegen, Geert Groteplein 10, 6525 GA, Nijmegen, The Netherlands; 4 School of Medical Sciences, Department of Health Systems Innovation and Education, VU University Medical Center, De Boelelaan 1117, 1081 HV, Amsterdam, The Netherlands

**Keywords:** Postgraduate medical education, Progress testing, Knowledge assessment, Internet access, Construct validity, Reliability, Face-validity

## Abstract

**Background:**

Yearly formative knowledge testing (also known as progress testing) was shown to have a limited construct-validity and reliability in postgraduate medical education. One way to improve construct-validity and reliability is to improve the authenticity of a test. As easily accessible internet has become inseparably linked to daily clinical practice, we hypothesized that allowing internet access for a limited amount of time during the progress test would improve the perception of authenticity (face-validity) of the test, which would in turn improve the construct-validity and reliability of postgraduate progress testing.

**Methods:**

Postgraduate trainees taking the yearly knowledge progress test were asked to participate in a study where they could access the internet for 30 minutes at the end of a traditional pen and paper test. Before and after the test they were asked to complete a short questionnaire regarding the face-validity of the test.

**Results:**

Mean test scores increased significantly for all training years. Trainees indicated that the face-validity of the test improved with internet access and that they would like to continue to have internet access during future testing. Internet access did not improve the construct-validity or reliability of the test.

**Conclusion:**

Improving the face-validity of postgraduate progress testing, by adding the possibility to search the internet for a limited amount of time, positively influences test performance and face-validity. However, it did not change the reliability or the construct-validity of the test.

## Background

The best way to assess knowledge in postgraduate medical education remains a topic of debate and research. This issue is further complicated by the fact that the role of memorized knowledge is shifting. With the emergence of the widespread availability of fast internet most doctors nowadays have continuous access to the internet during both their outpatient and inpatient duties [1-3]. It has become very common to search for information or check guidelines during or after a doctor-patient contact. At the same time the database of biomedical knowledge is expanding rapidly, increasing the importance of the internet as a quick source of up-to-date information. It is therefore questionable whether assessment of knowledge during postgraduate medical training should solely focus on testing memorized knowledge. Perhaps the internet should be seen as an ’extended memory’ to which trainees should be allowed access during postgraduate medical knowledge tests, making them more in line with modern clinical practice [4].

Recently we evaluated the utility of so-called knowledge progress testing in postgraduate medical education to find a limited construct validity and moderate reliability [5]. The progress test is a yearly formatively intended knowledge test. It is designed to measure the growth of a trainee’s functional knowledge level on items relevant to daily clinical practice over training years and generate feedback to optimize learning. Efforts to improve the psychometric characteristics of the test, by changing from true-false questions to multiple choice questions, placing more emphasis on knowledge questions that are relevant to daily clinical practice, and including 20% ‘old’ questions that have shown good discriminative power, improved the test’s reliability somewhat but did not have an effect on its construct-validity*.* Along with a limited construct validity and reliability, we found that trainees were discontented with the face-validity (trainees question whether the test measures the intended construct) and educational impact of the test [5,6]. Furthermore, educational supervisors and trainees indicated that they found the assessment of sheer memorized knowledge to be outdated, since there is now so much easily accessible, reliable and up-to-date information available online. The test’s perceived lack of authenticity, i.e. the test does not adequately reflect daily clinical practice, could explain its apparent lack of face-validity and educational impact.

As improving the authenticity of a test may well result in a better test-utility by affecting face-validity and/or reliability and construct validity [7-9], allowing time-restricted internet access during progress testing seems a reasonable step. During both in- and out-patient duties the knowledge of trainees is challenged continuously. They need to be certain of what they know and what they do not know, and where they can find accurate information quickly on the internet. To reflect the reality of daily practice, where time is not indefinitely available due to heavy service pressure and situations involving life-threatening emergencies [10,11], we limited the amount of time the internet could be accessed during the test.

To the best of our knowledge no previous studies have reported on the effect of allowing internet access during postgraduate knowledge testing. While there are several studies that address open-book testing, these are fundamentally different from the study we envisioned. First of all, during an open-book test the book is allowed during the entire exam. Furthermore, open-book tests are usually designed to evaluate understanding rather than recall and memorization [12-14]. The progress test, however, is designed to measure recall and memorization. In this situation, allowing internet access can be seen as allowing access to the ‘extended memory’ that is part of everyday clinical practice. For this reason we have designed a study to test the benefit of allowing internet access during the yearly knowledge progress test taken by postgraduate trainees.

### Purpose of the study

The aim of the study is to test whether having the option to access the internet for a restricted amount of time during postgraduate knowledge progress testing improves the face-validity, construct validity and/or reliability of postgraduate knowledge progress testing.

## Methods

### Progress test

The progress test, a national, yearly formative knowledge test attuned to graduate level, is an obligatory part of postgraduate training in Obstetrics and Gynecology (O&G) in the Netherlands. Postgraduate trainees in Obstetrics and Gynecology in the Netherlands can start at any moment during the year and the training year is determined by the date the trainee started. For example, a trainee that started 1 May 2007 will be in his/her second training year during the progress test of April 2009. Furthermore, trainees have variable backgrounds regarding their previous clinical experience. The test consists of 150 multiple-choice questions divided over 5 subdomains according to a pre-defined blueprint. Every year the progress test exam committee, consisting of 10 practicing gynecologists who each represent one or more subdomains, devises a completely new set of questions. The focus of the test is on knowledge that is relevant to daily clinical practice for a newly graduated gynecologist. A question mark is included in the answer options to discourage from guessing. All trainees take the test simultaneously and test time is limited to 180 minutes. The test is norm referenced; test results are reported as the number of correct answers minus incorrect answers percentage score per training year. Using the question mark option is not punished. As the test is intended to be used formatively (to provide feedback that helps direct learning), there are no consequences for failing the test.

### Participants and procedures

In 2009 all eight medical schools that provide postgraduate training in O&G in the Netherlands were asked whether they were willing and able to grant internet access during the yearly progress test. Six out of the 8 medical schools responded positively. All 192 trainees of these six medical schools were approached with a letter, asking them to take part in a study that would allow them internet access during part of the progress test. They were asked to give written consent.

The national progress test in O&G was taken by 259 trainees in 2009 (see flow chart). One hundred and ninety two trainees had been approached beforehand to participate in the study, of which 161 (83%) consented to take part. This left 97 trainees who only sat the traditional, pen and paper the test and 1 trainee who dropped out because of maternity leave.

Of the 161 participants, six did not hand in the second answer sheet, possibly because they left the exam room early. Two participants did not clearly indicate which answers they changed after accessing the internet and were excluded, leaving 153 participants whose data were complete and suitable for analysis.

### Study design

The study was set up as a before and after intervention study. The yearly progress test of 2009 consisted of two parts: a traditional, pen and paper test for all trainees, followed by the possibility for participating trainees to use the internet for 30 minutes. To produce norm-referenced test scores, both participating and non-participating trainees were allowed 150 minutes to finish the traditional part of the test. During this time, participants in the study were advised to note their answer choices in the question booklet to allow for changes later. At the end of the traditional part of the test, all trainees were asked to hand in their answer sheets, and trainees not participating in the study left the exam room. Next, trainees in the study group were granted 30 minutes of internet access and were instructed to note only the answers they wanted to change on a second answer sheet. This study design was chosen as we were concerned that allowing participants to start up the internet as soon as they finished the traditional part might disturb other candidates. On the other hand, many trainees do not need the full 180 minutes to finish the test, and we were afraid that participants would not want to stay for the part with internet access if they had to wait too long.

We designed a short pre- and post-test questionnaire to explore the effect of internet access on the face-validity of the test. The questionnaires could be completed anonymously online two weeks before and one week after the knowledge test. The pre-test questionnaire consisted of two statements regarding the face-validity of the traditional test that could be answered by a 5-point Likert Scale: 1 strongly disagree, 2 disagree, 3 neutral, 4 agree, and 5 strongly agree. The post-test questionnaire consisted of five statements that could be answered in a similar fashion: two of the questions were on the face-validity of the test with internet access, two questions were on test time, and a final question was on whether internet access should continue to be allowed during testing.

**Figure 1:**
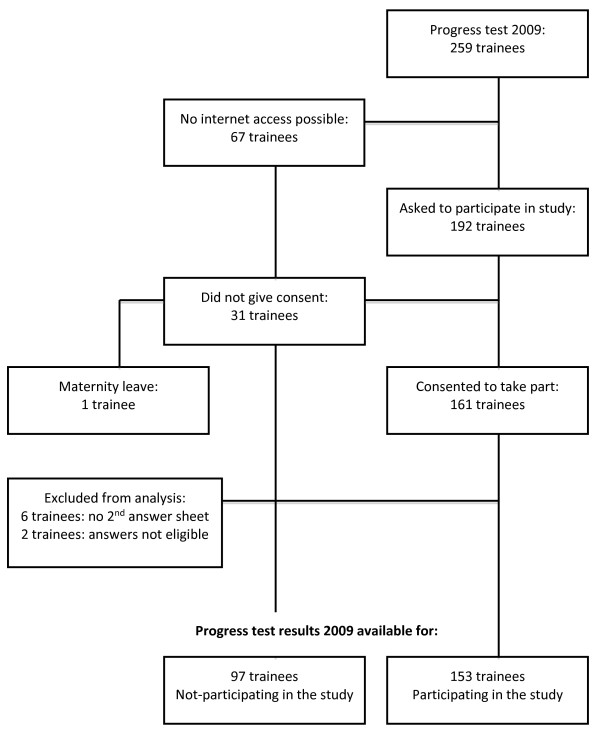
Flow chart in- and exclusion.

Of the 161 trainees participating in the study, 98 (61%) completed the online pre-test questionnaire and 83 (52%) completed the post-test questionnaire. The content of the questionnaire is reported along with the participants’ answers in the results section. As the questionnaires were answered anonymously, we were not able to distinguish between trainees who answered both questionnaires and those who did not (Figure 1).

### Data analysis

All answer sheets were computer read into SPSS 19.0, as were the answers on the online questionnaires.

Instruments:

● Progress test results before internet access (PT)

● Progress test results after internet access (PT+)

● Questionnaire before internet access

● Questionnaire after internet access

Data were analyzed for:

● Effect size with the repeated measures ANOVA

● Significant differences in mean score per training year before and after internet access with the paired samples t-test

● Differences in ranking of test scores before and after internet access by Wilcoxon signed ranks test

● Construct validity, or knowledge growth, by comparing training year means by one-way Anova and post-hoc Scheffé’s test both before and after internet access

● Reliabity with Cronbach’s α

● Significant differences in pre and post-test questionnaires answers with the independent samples t-test

### Ethical considerations

At the time of this study there was no formal ethical review committee for medical education research in the Netherlands, and the local IRB ruled that this type of research is exempt. However, we took great care to inform all participants about the purpose and voluntary nature of the study before asking for their written consent.

## Results

A comparison of mean test scores of participating and non-participating trainees before internet access was allowed shows significantly lower test scores for non-participating trainees in training year 2 and 3. This suggests that the group of participants is not a representative sample of the total group of trainees. However, when the mean scores of non-participating trainees are split into two groups: trainees who were invited to take part, but declined, and trainees that were not invited because their medical school could not provide internet access during the test, there is no clear trend distinguishable which group performed worst (Table 1).

**Table 1:** Mean percentage progress test scores for trainees that participated in the study and those that did not (before internet access)

**Training**	**N**	**Part**^**1**^	**N**	**Not part**^**2**^	**p**	**Invited**^**3**^		**Not invited**^**4**^	
**Year**		**Mean**		**Mean**		**N**	**Mean**	**N**	**Mean**
**1**	28	25.6	15	18.7	0.78	2	14.9	13	19.3
**2**	27	30.1	18	22.1	0.002	6	25.8	12	20.3
**3**	32	36.2	15	27.8	0.02	4	25.2	11	28.7
**4**	16	30.2	19	33.4	0.49	9	33.2	10	33.7
**5**	28	37.2	12	35.0	0.52	2	29.5	10	36
**6**	22	30.8	18	34.4	0.22	7	31.8	11	36
**Total**	153		97			30		67	

^1^Part: participating in the study.

^2^Not part: not participating in the study.

^3^Invited: invited to participate, but declined.

^4^Not invited: 2 universities that were not able to grant internet access during progress test.

On average the answer to 8.4 (range 0–18) or 6% of the PT + questions was changed during the 30 minutes of internet access. An average of 6.7 (range 0–18) of these questions were changed to a correct answer, and 1.7 (range 0–9) to an incorrect answer. As a result, test scores for every training year increased after internet access. Table 2 shows the mean PT and PT + scores per training year. Repeated measures ANOVA showed that the difference between the mean PT and PT + scores was statistically significant (*F*(1, 18) = 979,035, *P* = 0.000). The partial eta-squared (η^2^ = .55) was of medium size. During the internet access period all of the questions’ answers were changed by at least one trainee. In fact, the number of answer changes ranged from 3 to 53, depending on the question. Answers were changed most often for questions concerning facts or percentages or were initially answered by a question mark. The answers to more practically orientated questions were only occasionally changed.

**Table 2:** Mean percentage progress test scores per year group before (PT) and after (PT+) internet access

**Training year**	**N**	**PT**	**PT+**	**p**
		**Mean (sd)**	**Mean (sd)**	
**1**	28	25.6 (11.8)	29.5 (13.0)	< 0.05
**2**	27	30.1 (8.5)	33.0 (8.8)	< 0.05
**3**	32	36.2 (10.7)	39.5 (10.9)	< 0.05
**4**	16	30.2 (13.0)	34.0 (12.6)	< 0.05
**5**	28	37.2 (10.9)	40.1 (11.1)	< 0.05
**6**	22	30.8 (6.6)	35.9 (8.3)	< 0.05
**Total**	153			

The Wilcoxon signed ranks’ test showed a significant difference in ranking before and after internet access. The ranking table is not included in the manuscript but can be obtained from the authors.

### Construct validity

**Figure 2:**
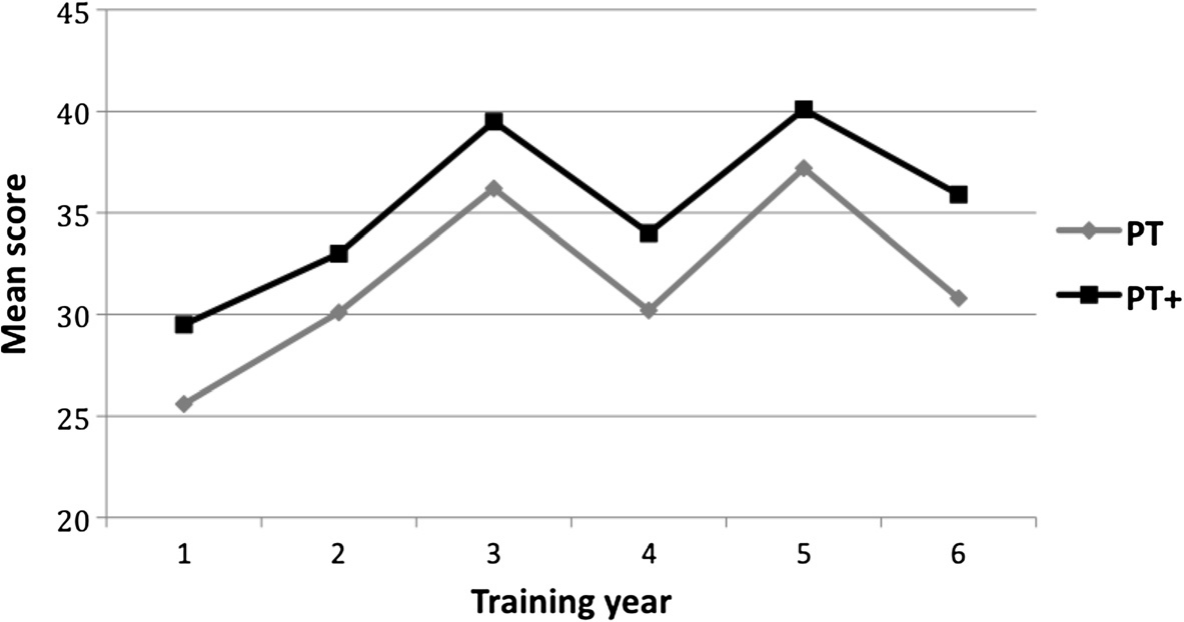
Progress test scores before and after internet access.

Statistical analysis of mean test scores per training year using one-way Anova followed by post-hoc Scheffé’s test shows that the only significant difference was between the test scores of training year 1 and 2 and higher training years (Figure 2). Adding internet access does not influence this finding. The tables are not included in the manuscript but can be obtained from the authors.

### Reliability

Table 3 shows Cronbach’s alpha for both the PT and the PT + for every training year. For the closed-book part, Cronbach’s alpha ranged from.355 to .821; after internet access Cronbach’s alpha ranged from .552 to .831.

**Table 3:** Cronbach’s alpha

**Training year**	**PT**	**PT+**
**1**	.821	.831
**2**	.631	.553
**3**	.703	.705
**4**	.747	.690
**5**	.702	.716
**6**	.355	.588

### Face-validity

The mean Likert scale scores for the questions regarding face-validity are presented in Table 4. On a 5-point scale trainees indicated that the progress test with internet access more acurately reflected the knowledge needed as a gynecologist (3.2 vs. 2.3 p < 0.05) and that the progress test with internet access is a better instrument to test their knowledge level (3.2 vs. 2.4 p < 0.05).

**Table 4:** Pre test and post test questions on face-validity

**Question**	**Mean (sd)**	**Mean (sd)**	**Significance**
	**pre test**	**post test**	
The *traditional* progress test appropriately reflects knowledge required as a gynecologist*.	2.3 (0.99)		
	(n = 96)		
The*l* progress test *with internet access* appropriately reflects knowledge required as a gynecologist*.		3.2 (1.29)	p < 0.05
		(n = 82)	
The *traditional* progress test is a good instrument to test my knowledge level*.	2.4 (0.98)		
	(n = 94)		
The progress test *with internet access* is a good instrument to test my knowledge level*.		3.2 (1.12)	p < 0.05
		(n = 82)	

*1 = disagree completely, 2 = disagree, 3 = neutral, 4 = agree, 5 = agree completely.

Pre-test questionnaire was completed by 98 participants (61%).

Post-test questionnaire was completed by 83 participants (53%).

Table 5 contains the results post-test questions on the evaluation of internet access during knowledge progress testing. Most respondents (56%) would like the availability of internet access to continue, however, they would like more time to access the internet (95%).

**Table 5:** Post test questions evaluating internet access during the progress test

	**Disagree completely**	**Disagree**	**Neutral**	**Agree**	**Agree completely**
There was enough time to finish the part **without** internet access. (n = 81)	14%	11%	3%	50%	22%
There was enough time to finish the part **with** internet access. (n = 82)	84%	11%	3%	2%	0%
The *availability of* access during the progress test should be continued. (n = 82)	11%	11%	22%	42%	14%

Post-test questionnaire was completed by 83 participants (53%).

## Discussion

With this study we have shown that time-restricted internet access during knowledge progress testing does not improve the construct-validity or reliability of the test, even though it does increase face-validity and test scores for all training years. During 30 minutes of internet access trainees on average managed to change the answers to eight test questions, of which six were changed correctly and two incorrectly. A significant difference in ranking was demonstrated. However, the difference between the highest 20 scores and the lowest 20 scores is small and of questionable significance. The test’s reliability varied between year groups and ranged from .388 to .822, as measured by Cronbach’s alpha. Although in formative assessment an alpha of 0.65-0.75 is deemed acceptable [15], the test does not fulfill this criterion in 2 out of 6 measurements. However, face-validity, the extent to which the test is perceived by the trainees to reflect daily practice, does improve and most trainees would like the possibility of internet access during the progress test to continue, but they would like more time to access the internet.

### Construct-validity and reliability

Even though the face-validity of the test does improve, internet access does not improve construct validity or reliability as we had hypothesized. Regarding construct validity, we only found significant differences in mean test scores between lower training years. This finding had been reported by the authors and others previously [5,16]. The reliability per year group is inconsistent. This is probably due to the variable performance per year group, as can be deducted from the standard deviations in Table 2.

As this study is the first to research the potential benefits of internet access during postgraduate knowledge testing, we were not able to compare our findings to those of other studies. The most relevant literature addresses open-book testing in higher education. Several research groups have approached the issue of open-book testing from a similar background to ours: the ever-expanding online biomedical knowledge database, combined with the fact that in real-life situations resources usually are easily and freely available [13,17]. They have found that when an open-book test resembles a traditional knowledge test (like our progress test) no difference in test performance between open- and closed-book sections could be detected [17-19]. Little has been written regarding the influence of open-book testing on test validity and/or construct-validity. However, no difference in ranking can be demonstrated [4,17]. Regarding reliability, only a slight decrease in reliability was found by one research group, which they attributed to the ‘novelty’ of the test format [4]. Obviously there is a need for more research on the subject of the influence of the internet on both knowledge retention and knowledge test results [19,20].

### Face-validity

Face-validity, the degree to which the trainees find that the test measures the intended construct, appears to increase with the addition of internet access. This is in line with findings from studies concerning open-book tests, which students perceive as a better assessment of their knowledge relevant to modern clinical practice [4,19]. However, it also known that open-book tests provoke less test anxiety and that students tend to prepare themselves less for open-book tests [12-14,17]. We did not look into this aspect of internet access during the progress test, but it is possible that face-validity is not the only reason why our trainees favored having internet access. Furthermore, as our pre- and post-test questionnaires were anonymous, we were not able to analyze only the answers of those trainees that answered both questionnaires. Thus it is possible that we did not find a true effect.

The potential influence of face-validity on the psychometric characteristics of a test is heavily debated in the literature. Some argue that it constitutes an important aspect of the acceptability, and as such, utility of a test [8,21]. However, others consider it a garbage-can term that adds little or nothing to our understanding of assessment data [22]. Nevertheless, even though face-validity may not represent a robust psychometric construct, one can reason that the perceptions of stakeholders and the resulting level of acceptance of a test is an issue that deserves attention. This is especially true when evaluating formative assessment, which is intended to direct learning. If a test is not accepted as valid by trainees and/or supervisors, it is questionable whether the test’s results can have any impact on learning. In the utility formula proposed by van der Vleuten [8], acceptability and educational impact feature as important determinants of utility, next to traditional psychometric test characteristics such as validity and reliability [8,9].

### Time

Our study confirms that open-book type testing, which in our case involved searching the internet, costs time [19,23]. This is demonstrated by the fact that 30 minutes of internet access only allowed trainees to change an average of 8, predominantly factual, questions out of 150. In the post-test analysis trainees indicated that they would like to increase the amount of time that the internet can be accessed. However, it is questionable whether more internet time would change our findings for studies on open-book testing have shown an inverse relation between the amount of time spent looking things up and test results [14,19,24]. More time to access the internet would most certainly have a greater impact on psychometric test characteristics, though it is difficult to predict whether they will improve or deteriorate. Most importantly, instigating a time limit was part of the study design to increase the authenticity of the test, as time is also limited in real-life situations.

### Strengths and limitations

To our knowledge, this is the first study that explores the additional value of a limited period of internet access during a postgraduate knowledge test.

Our findings are somewhat limited by the fact that we did not randomize or control for this study and that the number of trainees per training year is small. In addition, we did not use a validated questionnaire to measure (changes in) face-validity. Furthermore, we did not study the effect of allowing internet access on the educational impact of progress testing, which is another major determinant of the utility of a formative test. As the study was conducted in the Netherlands, solely with postgraduate trainees in Obstetrics and Gynecology, the results may not be completely generalizable to other medical specialties or countries.

It can be argued that online access has no place in a knowledge test, because when the internet is used to search for information, rather than a reference book, a completely different skill is assessed. Nonetheless, finding reliable information on the internet can be quite an arduous task, as is demonstrated by our trainees, whose changes were incorrect a fourth of the time [25,26]. This makes internet search strategies and the interpretation of online information a skill worth assessing in postgraduate training.

## Conclusion

Improving the authenticity of postgraduate knowledge progress testing, by adding the possibility to search the internet for a limited amount of time, positively influences test performance and face-validity. However, it does not affect the reliability and/or construct-validity of the test.

Future research

● Does improving face-validity improve acceptability of formative knowledge assessment?

● Does improving face-validity improve educational impact of formative knowledge assessment?

● Explore other ways to increase construct-validity and reliability of formative knowledge assessment.

● Develop a strategy to assess internet search skills and interpretation of internet search results.

## Competing interests

The authors declare that they have no competing interests.

## Authors’ contributions

MD, FS, and IJ were involved in the study design. MD and IJ collected and analyzed the data. MD, FS and DB drafted and finalized the manuscript. All authors approved the final version.

## Pre-publication history

The pre-publication history for this paper can be accessed here:

http://www.biomedcentral.com/1472-6920/13/147/prepub
